# Effects of Semi-Immersive Virtual Reality-Based Cognitive Training Combined with Locomotor Activity on Cognitive Function and Gait Ability in Community-Dwelling Older Adults

**DOI:** 10.3390/healthcare9070814

**Published:** 2021-06-28

**Authors:** Na-Kyoung Hwang, Jong-Bae Choi, Dae-Kil Choi, Jae-Min Park, Chang-Wan Hong, Ji-Su Park, Tae-Hyung Yoon

**Affiliations:** 1 Department of Occupational Therapy, Seoul North Municipal Hospital, 38 Yangwonyeokro, Seoul 02062, Korea; occupation81@gmail.com; 2 Department of Occupational Therapy, Sangji University, 83 Sangjidae-gil, Wonju-si 26339, Korea; cjb3798@naver.com; 3 R&D Team, YOUCANSTAR Inc., 170 Gobun-ro, Yeonje-gu, Busan 47583, Korea; youcanstar@youcanstar.com (D.-K.C.); alsqkr1234@daum.net (J.-M.P.); cplus94@youcanstar.com (C.-W.H.); 4 Advanced Human Resource Development Project Group for Health Care in Aging Friendly Industry, Dongseo University, 47 Jurye-ro, Busan 47011, Korea; 5 Department of Occupational Therapy, Dongseo University, 47 Jurye-ro, Busan 47011, Korea

**Keywords:** older adult, virtual reality, cognitive function, gait

## Abstract

This study aimed to investigate the effects of semi-immersive virtual reality-based cognitive training (VRCT) combined with locomotor activity on cognitive function, balance, and gait ability in older adults. Eighteen community-dwelling older adults participated in this study. Subjects who met the selection criteria were assigned to an experimental group (*n* = 9) and a control group (*n* = 9). The experimental group received VRCT combined with locomotor activity for 30 min a day, three times a week, for 6 weeks. The control group received tabletop activity-based cognitive training for the same amount of time. Before and after the training, the Korean Mini-Mental State Examination (K-MMSE), Trail Making Test (TMT; A and B), and Digit Span Test (DST; forward and backward) were used to evaluate cognitive function; and the Timed Up and Go (TUG) test and 10-m Walking Test (10MWT) were used to evaluate the improvement in the balance and gait ability parameters. After the intervention, the experimental group showed a significantly greater improvement in the TMT-A (*p* = 0.045) and DST-backward (*p* = 0.012) scores compared with the control group. Regarding the gait ability variable, the experimental group showed a significant improvement in the 10MWT test (*p* = 0.001). This study confirmed that semi-immersive VRCT combined with locomotor activity is useful for improving cognitive function and gait ability in older adults. Therefore, VRCT combined with locomotor activity can be used as a simultaneous intervention for cognitive rehabilitation and functional capacity improvement in older adults.

## 1. Introduction

Aging in humans is usually accompanied by typical structural and neurophysiological changes in the brain and variable degrees of cognitive decline [1]. Cognitive frailty is emerging as one of the greatest health threats of the 21st century, and as life expectancy increases, the prevalence of cognitive decline and dementia is also increasing [2]. A previous study reported that the economic costs associated with dementia and other cognitive impairments in the United States ranged from $159 billion to $215 billion in 2010, and will increase to $510 billion by 2040 [3]. Therefore, cognitive rehabilitation approaches are important for maintaining and improving cognitive health and preventing further functional decline in older adults [4].

Aging is a major challenge for the healthcare system, especially with regard to the maintenance of functional capacity and independence, and the expansion of the framework of rehabilitation professionals [5]. Along with joint and lower limb muscle flexibility, balance and gait are major musculoskeletal factors that support functional capacity in older adults [6]. Gait instability and imbalance cause major public health problems such as falls and reduced social autonomy; therefore, they should be considered important for improving the health and quality of life of older adults [7]. 

With the advancements in information technology, virtual reality (VR)-based cognitive rehabilitation therapy [8,9] and VR games as resources for helping improve deteriorated functional capacity, have modernized the clinical practice of rehabilitation professionals [10,11]. VR allows users to experience and interact with computer-generated environments and to react as they would in real life when performing predetermined tasks [12]. This method can be used to perform fun and interesting tasks, thus increasing the user’s motivation [13]. VR ranges from non-immersive to immersive, depending on the degree to which users are isolated from the physical environment while interacting with the virtual environment [14]. In particular, immersive and semi-immersive VR systems have provided opportunities for motor or cognitive activities that cannot be implemented in a clinical environment by performing real-life scenarios and simulations of activities [15,16]. VR health applications have been conducted in several studies to address health-related issues in older adults [17]. VR application for older adults should be considered not only for its effectiveness, but also for the side effects of body reactions following VR application and VR acceptance. The use and development of immersive VR using head-mounted display (HMD) is expected to become more realistic and to promote generalization to real-life performance [18]; however, the negative attitudes of elderly users toward HMD VR and cybersickness could hinder this [19]. Semi-immersive VR has higher user immersion than non-immersive VR as it uses real images in a virtual environment and has fewer side effects, such as cybersickness [20]. For this reason, the use of semi-immersive VR in the field of rehabilitation is recommended. Semi-immersive VR has been demonstrated to be an effective intervention for improving balance and gait ability, as well as cognitive function in various subjects, including community-dwelling older adults and patients with neurological disorders [21,22].

The recently developed semi-immersive VR-based cognitive training (VRCT) combined with locomotor activity uses a three-sided projection surrounding the user, and allows the user to perform cognitive tasks by touching the screen directly. The program of this equipment addresses various cognitive components, such as visuospatial perception, memory, learning, attention, and decision making, and presents challenging game tasks to users. The user performs the cognitive game tasks projected in the three screens while moving their body dynamically. Most VR programs that combine physical activity and games are intended to perform specific physical movements in the field of motor learning [23]. However, VR programs equipped with complex tasks requiring cognitive and motor demands for older adults are rare. Therefore, this study aimed to provide evidence for the effect of semi-immersive VRCT combined with locomotor activity on cognitive function, balance, and gait ability in community-dwelling older adults.

## 2. Materials and Methods

### 2.1. Subjects

A total of 18 community-dwelling older adults were recruited into the study. The inclusion criteria were as follows: (1) age greater than 65 years; (2) no limitation in the range of motion of the upper and lower extremities; (3) a fair grade or higher in manual muscle testing of the upper and lower extremities; (4) independent performance of activities of daily life; (5) ability of appropriate communication; and (6) understanding of and conformance with instructions, methods, and procedures. The exclusion criteria were as follows: those with neurological history, unstable medical problems, history of psychiatric disorders, visual or auditory function problems, or severe communication difficulties. 

### 2.2. Study Design and Procedures

**Figure 1:**
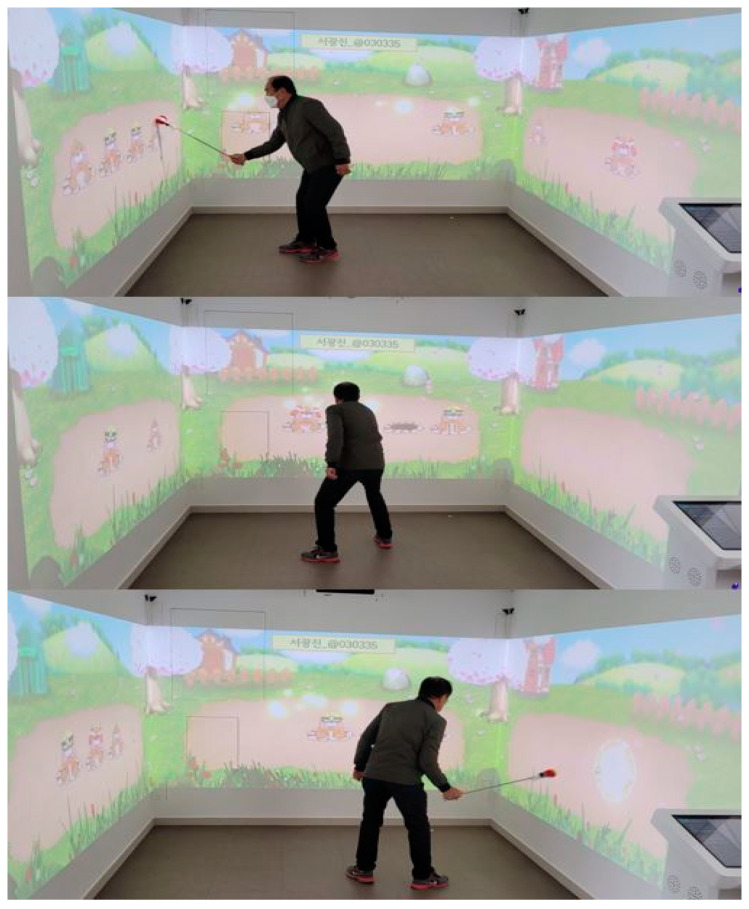
Virtual reality-based cognitive training combined with locomotor activity.

Twenty subjects were assigned to either the experimental or the control groups and completed a 6-week intervention. The experimental group performed locomotor activity-based cognitive training using the DoveConsol (YOUCANSTAR Inc., Busan, Korea; Figure 1). The DoveConsol consists of software for cognitive training, a large screen, and a beam projector. Cognitive training was provided through a large screen installed on the wall, and as the entire screen can be touched, it provides assistance in walking and balance training of the lower limbs during cognitive training. The cognitive program consists of shopping, puzzles, and mole catching to improve memory, concentration, and problem-solving skills. Training was carried out for 30 min a day, three times a week, for 6 weeks. Meanwhile, the control group performed tabletop activities, including puzzles, wood blocks, card play, construction activity, maze, and a pencil/paper activity for problem solving. The training schedule was applied in the same way as in the experimental group.

### 2.3. Outcome Measurements

To evaluate cognitive function, the Korean Mini-Mental State Examination (K-MMSE), Trail Making Test (TMT), and Digit Span Test (DST) were used. The K-MMSE is a brief screening test that quantitatively assesses cognitive status, and is tested for validity and efficacy in clinical settings [24]. It has a total score of 30 points and includes the following six categories: time orientation, spatial orientation, memory registration, attention and calculation, memory recall, language, and space–time configuration. The TMT was used to test the maintenance of attention and cognitive flexibility. It is a timed neuropsychological test that focuses on visual scanning, divided attention, and psychomotor speed [25]. The TMT consists of two parts, as follows: in part A, the subject is asked to connect randomly distributed numbers on the test paper in ascending order (1–2–3, etc.); in part B, the subject is required to alternate numbers and letters (1–A–2–B etc.). Part B requires more complex cognitive functions and has been proven to be sensitive to prefrontal cortical dysfunction [26]. It has a test–retest reliability of r = 0.78 and an inter-rater reliability of r = 0.99 [27]. The DST is a number memorization test used to evaluate working memory and attention to auditory stimuli. The subject hears a series of numbers and is required to recall the sequence correctly, with increasingly longer sequences being tested in each trial. In the DST-forward, the subject is asked to recall the numbers in forward order. It consists of three sets of numbers with lengths ranging from three to eight digits. On the other hand, DST-backward is a test that involves recalling the numbers in reverse order, which consists of three sets of numbers with lengths ranging from two to seven digits. The subject’s digit span is the longest consecutive digit that can be accurately recalled [28].

For balance and gait ability evaluation, the 10-m Walking Test (10MWT) and Timed Up and Go (TUG) test were used, respectively. The 10MWT measures the locomotor capacity in clinical and research settings, and the outcome measures the time taken to complete the test [29]. A subject walks a total of 13 m and is required to walk at ordinary walking speed from the start point to the end point. The walking time is measured, except for the 1.5 m predetermined from the starting point and the arrival point. The 10MWT has demonstrated excellent reliability in many conditions, such as Parkinson’s disease, hip fracture, spinal cord injury, stroke, and traumatic brain injury [30], as well as in healthy adults. In the TUG test, which measures dynamic balancing ability and functional mobility, a subject rises from an armrest chair, walks a 3-m distance, returns, and sits back on the chair. The TUG test was performed three times, and the average time needed to do the task was used. The TUG test takes an average of 7 to 10 s for healthy and normal older adults. Times in excess of 30 s are known to indicate dependent mobility or impairment to independently perform outdoor exercise [31].

### 2.4. Statistical Analysis

All of the statistical analyses were performed using SPSS version 15.0 (IBM Corp., Armonk, NY, USA). Descriptive statistics are presented as means with standard deviations. The Shapiro–Wilk test was used to check for normality of the outcome variables. To evaluate the training effects, a Wilcoxon signed rank test was used to compare the measures before and after the intervention in each group. The Mann–Whitney U test was used to compare post-intervention values and changes in outcome measures between the two groups. In addition, the effect sizes (Cohen d) of the changed scores between the two groups were calculated. Effect sizes of 0.2, 0.5, and 0.8 represent small, moderate, and large effects, respectively.

## 3. Results

### 3.1. Subjects’ Characteristics

Eighteen older adults were enrolled in this study, and there was no dropout until the intervention was completed. Therefore, the data from 18 people were analyzed (Table 1). There were no significant differences between the groups based on general characteristics and the MMSE (*p* = 0.732), TMT-A (*p* = 0.235), TMT-B (*p* = 0.275), DST-forward (*p* = 0.32), and DST-backward (*p* = 0.80) scores. 

### 3.2. Cognitive Function Evaluation

Based on within-group comparisons (pre-training vs. post-training), the experimental groups showed statistically significant improvements in the TMT-A (*p* = 0.012) and DST-backward (*p* = 0.025). In contrast, the control group had no significant increase in all of the assessments. After the intervention, the experimental group had significant improvements in the TMT-A (*p* = 0.045), and DST-backward (*p* = 0.012) results compared with the control group (Table 2). Cohen’s d effect size was 0.82, 0.02, 0.48, and 0.60 for the TMT-A, TMT-B, DST-forward, and DST-backward, respectively.

### 3.3. Gait and Balance Evaluations

Based on within-group comparisons (pre-training vs. post-training), the experimental group showed statistically significant improvements in the 10MWT (*p* = 0.028). In contrast, there was no statistically significant difference in any of the items in the control group. After the intervention, the experimental group showed a statistically significant improvement in the 10MWT compared with the control group (*p* = 0.0001; Table 2). Cohen’s d effect size was 0.3 and 1.4 for the TUG test and DST-backward, respectively.

## 4. Discussion

This study investigated the effectiveness of semi-immersive VRCT combined with locomotor activity at improving cognitive function, balance, and gait ability in community-dwelling older adults. Our results showed that VRCT combined with locomotor activity was more effective than conventional therapy for cognitive function (not balance and gait ability) at improving certain aspects of complex attention, capacity of working memory in cognitive function, and gait speed in the locomotor ability of the subjects.

It has been proven through several studies that the interaction between humans and VR improves cognitive function [32,33]. Gamito et al. [34] suggested the positive effects of VR cognitive stimulation on general cognition, executive function, attention, and visual memory, and the results of these improvements improved executive function. The findings of this study showed that VRCT improved the attention and capacity of working memory, and previous studies support the results of this study. This can be achieved by the biological effects of the activation of neuroplasticity, such as enhancing or attenuating synaptic transmission [35] and the remodeling of synaptic connections [36]. The cognitive performance did not improve in the control group receiving the tabletop activity-based cognitive training. The VR cognitive tasks were provided in a virtual environment, including shops and at home, and required problem solving through the interaction of multiple cognitive components. On the other hand, most of the stimuli for the control group who received conventional cognitive training was derived from pencil/paper or tabletop activities, primarily aimed at improving a single cognitive component. The lack of improvement in the cognitive performance of the control group may be due to their lack of experience in memory strategy implementation and mental flexibility. Several previous studies on VR-based cognitive training have demonstrated similar results [37,38].

Recently, interventions that combine cognitive and motor challenges have applied specialized equipment and/or technology that allow simultaneous access to two training elements [39,40,41]. Some studies have suggested that interventions that combine cognitive and motor challenges can improve gait and cognitive performance [42,43]. Physical activity has positive metabolic effects by increasing the levels of the neurotrophic factors derived from the brain and blood flow to the hippocampus [44]. This enhances the neuroplasticity potential of the brain and accelerates the learning process while performing tasks [45]. In particular, stimulation of the hypothalamic–pituitary–adrenal axis by physical activity increases cortisol levels and improves memory and learning [46]. Thus, interventions combined with cognitive and motor challenges have shown promise; in this respect, our study provides evidence for this.

A change in balance is a major factor affecting falls in older adults [47], and dynamic balance exercises, in particular, can significantly reduce the fall risk factor [48]. VR-based exercise programs can improve anticipatory postural adjustments, postural responses, sensory orientation, and balance during gait in older adults. The VRCT program in this study requires simultaneous continuous gross motor activity of the upper and lower extremities. The VRCT group showed a greater increase in TUG time than the CT group, confirming that VRCT combined with locomotor activity is effective at improving postural balance. Previous studies corroborate the present results. Cikajlo et al. [49], who studied remote rehabilitation using VR-based exercise tasks, reported improved TUG times after intervention. Karahan et al. [50] reported the effectiveness of virtual games with Xbox Kinect compared with home-based balance training on a PC for functional mobility and quality of life.

Older adults tend to increase stability by decreasing walking speed and shortening the stride length while increasing the time spent in double-support stance [51]. Games for gait training include the ability to transfer body weight between limbs, triple flexion (hip, knee, and ankle), one-leg support during mid-terminal stance, and load acceptance during initial contact [52]. VRCT is composed of tasks to improve cognitive function, which requires continuous weight movement and the execution of gait-related variables to perform cognitive tasks in a standing position. As a result of this study, the VRCT group showed a significant increase in velocity, revealing an improved gait ability. Adequate gait speed is an essential factor for independent outdoor ambulation and mobility in satisfactory communities [53]. Therefore, VRCT can be an effective approach for improving the gait ability of older adults.

This study has some limitations. First, the small sample size of this study, as a result of limited resources, may decrease the statistical power and generalizability; thus, future studies, given sufficient resources, should consider implementing a large-scale intervention. Second, there was no evaluation of the subjects’ motivation, acceptance of VR technology, or adverse reactions such as cybersickness and dizziness. Further research is needed on the psychological factors that promote further training and the factors that hinder training. Third, this study only proved the effects after 6 weeks of training, but it could not predict the sustained effect of the intervention. Investigation of the long-term impact of interventions on cognitive function, balance, and gait in the future is necessary. Fourth, hemodynamic changes such as heart rate, which could be evidence of the intervention intensity of VRCT combined with locomotor activity, were not measured; future interventions should include parameters to quantitatively measure changes in the hemodynamic biosignal. Finally, the current VR system lacks ecological validity in terms of portability and cost effectiveness for use in homes and rehabilitation clinics; thus, it is necessary to develop an advanced VR system with minimal equipment size and space requirements that guarantees sufficient physical activity, a simplified installation procedure, and a reasonable cost for private practice, in order to improve cognition and locomotor function in older adults.

## 5. Conclusions

This study confirmed that semi-immersive VRCT combined with locomotor activity is useful for improving certain aspects of complex attention, capacity of working memory in cognitive function, and gait speed in locomotor ability in older adults. Therefore, VRCT combined with locomotor activity can be used as a simultaneous intervention for cognitive rehabilitation and functional capacity improvement in older adults. However, further studies are needed on clinical samples of older adults to confirm the effectiveness in cognitive function and gait ability, stable maintenance through follow-up assessments, and the acceptance of this VR intervention.

## Figures and Tables

**Table 1:** Demographic characteristics of the patients.

	Experimental Group (*n* = 9)	Control Group (*n* = 9)
Number of subject	9	9
Gender (man/woman)	4:5	5:4
Age (year)	70.1 ± 3.9	69.2 ± 4.1
Educational level		
Uneducated	1	1
Elementary school	2	2
Middle School	4	4
High school	1	1
University	1	1

**Table 2:** Comparison of results between the experimental group and control group.

	Experimental Group				Control Group				Between Groups *p*-Values
	Before Treatment	After Treatment	Mean Difference	*p*-Value	Before Treatment	After Treatment	Mean Difference	*p*-Value	
K-MMSE	25.90 (1.79)	27.00 (2.30)	1.10 (2.13)	0.131	25.90 (0.99)	26.60 (1.07)	0.70 (0.82)	0.055	0.393
TMT-A	57.54 (19.31)	49.36 (11.18)	−8.18 (13.84) ^†^	0.012 *	56.40 (11.45)	58.00 (12.65)	1.60 (5.05)	0.357	0.045 ^†^
TMT-B	204.70 (65.48)	207.60 (48.00)	2.90 (59.92)	0.721	204.50 (44.01)	200.60 (36.52)	−3.90 (12.71)	0.359	0.791
DST-Forward	3.90 (1.28)	4.50 (1.50)	0.60 (1.17)	0.131	3.70 (0.67)	3.70 (0.94)	0.00 (0.66)	1.000	0.274
DST-Backward	2.50 (0.70)	3.00 (0.66)	0.50 (0.52)	0.025 *	2.60 (0.51)	2.70 (0.67)	0.10 (0.56)	0.564	0.012 ^†^
TUG	7.27 (2.81)	7.78 (1.57)	0.51 (2.64)	0.445	8.23 (1.15)	8.14 (0.85)	−0.09 (0.66)	0.506	0.705
10MWT	7.98 (1.97)	6.27 (0.77)	−1.71 (1.92)	0.028 *	8.57 (1.12)	8.00 (0.87)	−0.57 (1.15)	0.086	0.001 ^†^

K-MMSE—Korean Mini-Mental State Examination; TMT—Trail Making Test; DST—Digit Span Test; TUG—Timed Up and Go; 10MWT—10-m Walking Test; * *p* < 0.05 for the Wilcoxon signed rank test; ^†^ *p* < 0.05 for the Mann–Whitney U test.
